# Antibiotic-Associated Drug Reaction With Eosinophilia and Systemic Symptoms (DRESS) in a Young Male: A Case Report of Recurrence Without Organ Involvement

**DOI:** 10.7759/cureus.98963

**Published:** 2025-12-11

**Authors:** Mustafa Al Hassani, Zaid Al Hassani, Dhiaalden Al Amri, Alaa Al Masri, Razan Darwish, Aya Shubbar, Aqeel Saleem, Muhammed Rashid

**Affiliations:** 1 Internal Medicine, Sheikh Tahnoon Medical City, Al Ain, ARE; 2 Medicine, Sheikh Tahnoon Medical City, Al Ain, ARE; 3 Medicine, Al Kuwaiti Hospital, Sharjah, ARE; 4 Infectious Disease, Sheikh Tahnoon Medical City, Al Ain, ARE; 5 Internal Medicine, Sheikh Tahnoon Bin Mohammed Medical City, Al Ain, ARE

**Keywords:** antibiotic hypersensitivity, autoimmunity, dress syndrome, regiscar criteria, severe cutaneous adverse reaction (scar)

## Abstract

Drug reaction with eosinophilia and systemic symptoms (DRESS) is a rare but severe idiosyncratic hypersensitivity reaction, most commonly associated with antiepileptics and allopurinol. Although antibiotics are increasingly recognized as triggers, they remain underreported. The syndrome is characterized by fever, rash, eosinophilia, and variable organ involvement, with mortality.

We report the case of a 26-year-old previously healthy male who presented with acute fever, diffuse morbilliform rash, facial edema, and eosinophilia following ceftriaxone exposure. His symptoms improved with corticosteroids and drug withdrawal, but he experienced a relapse shortly after starting Helicobacter pylori triple therapy. The second episode resolved with discontinuation of antibiotics and supportive care. Importantly, there was no renal, hepatic, or cardiac involvement in either episode, and the diagnosis of DRESS was confirmed with a RegiSCAR score of 3.

This case highlights the variable presentation of DRESS, the diagnostic challenge posed by its overlap with other severe cutaneous adverse reactions, and the potential for relapse following re-exposure to structurally unrelated antibiotics. While the absence of systemic organ dysfunction contributed to a favorable outcome, the presence of positive autoantibodies underscores the importance of long-term follow-up due to the risk of autoimmune sequelae. Antibiotic-associated DRESS should be considered in patients presenting with rash, fever, and eosinophilia. Early drug withdrawal, structured diagnostic scoring, and long-term monitoring are essential to reduce morbidity and detect late complications.

## Introduction

Drug reaction with eosinophilia and systemic symptoms (DRESS) is a rare but severe idiosyncratic hypersensitivity reaction and one of the major severe cutaneous adverse drug reactions (SCARs). It is characterized by fever, widespread morbilliform rash, lymphadenopathy, eosinophilia, and potential multiorgan involvement, with mortality reported between 10% and 40% depending on the extent of systemic disease [1,2]. The incidence is estimated to range from 1 in 1,000 to 1 in 10,000 drug exposures [3]. Diagnosis is often challenging because of its delayed onset, usually three to eight weeks after drug initiation, and its overlap with other SCARs such as Stevens-Johnson syndrome and toxic epidermal necrolysis [4].

The pathogenesis of DRESS is incompletely understood, but several mechanisms have been proposed. These include genetic defects in drug detoxification pathways, such as polymorphisms in cytochrome P450 enzymes and N-acetyltransferases (NAT1 and NAT2), leading to accumulation of reactive metabolites, reactivation of latent viruses such as human herpesvirus 6 and 7, Epstein-Barr virus, and cytomegalovirus, as well as associations with certain HLA alleles and immune dysregulation [5,6].

The clinical presentation is variable. While the classic triad consists of fever, rash, and eosinophilia, organ involvement is common and most frequently affects the liver and kidneys, though the pancreas, thyroid, heart, nervous system, and other organs may also be implicated [2,7]. The latency from drug initiation to symptom onset typically ranges from several weeks, but it can be shorter in some antibiotic-related cases and on re-exposure, and resolution after drug withdrawal is often protracted over several weeks [2,7]. In some patients, the clinical picture may evolve or even become apparent only after the offending medication has been discontinued, reflecting the delayed and self-sustaining nature of the immune response. A wide range of medications has been implicated in DRESS. Antiepileptics such as carbamazepine, lamotrigine, and phenytoin account for about one-third of cases, allopurinol and sulfonamides are also frequent culprits, and antibiotics, including vancomycin and beta-lactams, are increasingly recognized as important triggers [4,8]. Although antiepileptics and allopurinol remain the most frequently reported culprits, antibiotics now represent a substantial minority of cases in contemporary series, underscoring the growing clinical relevance of antibiotic-associated DRESS. Within this group, beta-lactam antibiotics appear to be less commonly described than some other classes, which makes ceftriaxone-associated DRESS particularly notable.

Management begins with prompt recognition and immediate discontinuation of the offending agent [9]. Systemic corticosteroids are considered the mainstay of therapy, while other immunosuppressive or adjunctive treatments, including intravenous immunoglobulin, cyclosporine, mycophenolate mofetil, plasmapheresis, and antiviral therapy, have been used in severe or refractory cases [4,10]. Because relapse and long-term autoimmune sequelae are possible, careful follow-up and ongoing monitoring are recommended [7]. Recent national and expert guidelines provide structured recommendations for the diagnosis, risk stratification, and management of DRESS, emphasizing standardized assessment and long-term follow-up [11].

Although antiepileptics and allopurinol account for most reported cases, antibiotics are increasingly recognized as important triggers. Here, we describe the case of a 26-year-old man who developed DRESS following exposure to multiple antibiotics, with recurrence after re-exposure to a different antibiotic regimen. Uniquely, as demonstrated in this case, even patients with extensive cutaneous involvement may present without renal, hepatic, or cardiac dysfunction. Highlighting this atypical phenotype is important, as the absence of visceral organ damage can delay recognition and underscores the need for early clinical suspicion, prompt drug withdrawal, and close follow-up.

## Case presentation

**Figure 1:**
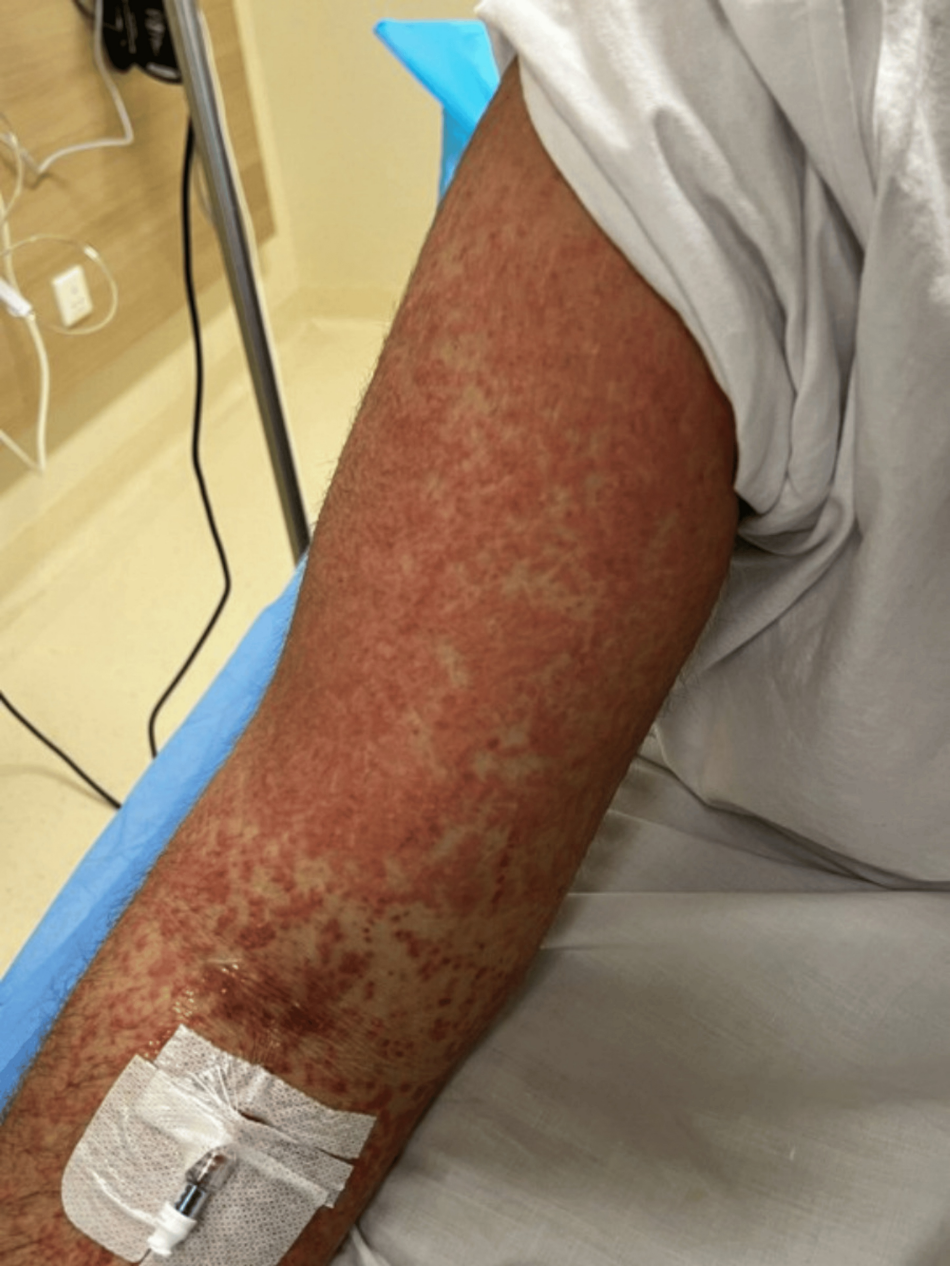
Generalized pruritic maculopapular eruption on the forearm. Erythematous, confluent maculopapular exanthem over the forearm at presentation. Similar lesions were present on the posterior neck, upper and lower limbs, chest, abdomen, genitals, and back.

A 26-year-old previously healthy male presented to our emergency department with a one-day history of acute fever and a generalized rash. The fever had reached 38.5 °C and was associated with a rapidly spreading pruritic eruption that began over the posterior neck and extended to the upper and lower limbs, chest, abdomen, genitals, and back (Figure 1). He also described mild facial swelling and a dry cough. There was no history of drug or food allergy, and he denied chest pain, wheezing, shortness of breath, palpitations, hemoptysis, orthopnea, abdominal pain, urinary changes, sore throat, headache, or arthralgia. He had no prior surgeries, no significant family history, and was not taking any regular medications.

The patient had been admitted one month earlier for presumed meningoencephalitis, treated empirically with a two-week course of intravenous ceftriaxone, vancomycin, and acyclovir. Around the tenth day of that admission, he developed a transient, mildly pruritic rash confined to the palms, which resolved with topical betamethasone and oral desloratadine. No lumbar puncture was performed at that time.

On the current illness onset (Day 0), while traveling in Oman, he sought care at a private clinic where he received an intramuscular antihistamine injection, reported to be diphenhydramine, before being referred to our hospital. Upon arrival at the emergency department, he was febrile at 38.5 °C, hypotensive with a blood pressure of 95/60 mmHg, tachycardic with a heart rate of 110 beats per minute, and his oxygen saturation was 98% on room air. He was started on intravenous ceftriaxone, and shortly afterward, his rash worsened dramatically, accompanied by recurrent fever and severe pruritus. Ceftriaxone was immediately discontinued, and he was treated with 300 mg of intravenous hydrocortisone over 10 hours and 50 mg of intravenous diphenhydramine.

On examination, he appeared acutely ill with a diffuse confluent morbilliform eruption and excoriations involving the trunk and extremities. The rash was accompanied by mild periorbital swelling. There were no mucosal erosions, no palpable lymphadenopathy, and no hepatosplenomegaly. Cardiopulmonary and abdominal examinations were otherwise unremarkable. At this stage, the differential diagnosis included viral exanthem, drug hypersensitivity reaction, and other severe cutaneous adverse reactions such as Stevens-Johnson syndrome/toxic epidermal necrolysis or serum sickness-like reaction. However, the absence of mucosal erosions, together with the temporal association with recent antibiotic exposure and the subsequent clinical course, made DRESS the most likely diagnosis and argued against Stevens-Johnson syndrome/toxic epidermal necrolysis or a primary infectious process.

Laboratory investigations on admission revealed leukocytosis with neutrophilia and eosinophilia, mild hepatic enzyme elevation, elevated inflammatory markers, and hypoalbuminemia. The results are summarized in Table 1.

**Table 1:** Initial laboratory values on admission. Laboratory findings at initial presentation demonstrated leukocytosis with neutrophilia and eosinophilia, mild elevation of alanine aminotransferase, hypoalbuminemia, and markedly raised inflammatory markers (CRP and procalcitonin). Renal function, cardiac enzymes, and BNP were within normal limits. WBC, white blood cell count; eGFR, estimated glomerular filtration rate; AST, aspartate aminotransferase; ALT, alanine aminotransferase; ALP, alkaline phosphatase; CRP, C-reactive protein; BNP, B-type natriuretic peptide

Test	Result	Normal range
WBC	15.2 × 10⁹/L	4.0-11.0 × 10⁹/L
Neutrophils	12.46 × 10⁹/L	2.0-7.0 × 10⁹/L
Eosinophils	1.16 × 10⁹/L	0.02-0.5 × 10⁹/L
Creatinine	77 µmol/L	60-110 µmol/L
Urea	4.9 mmol/L	2.5-7.5 mmol/L
eGFR	133 mL/min	>90 mL/minute
AST	11 IU/L	10-40 IU/L
ALT	52 IU/L	7-55 IU/L
ALP	66 IU/L	40-120 IU/L
Albumin	33 g/L	35-50 g/L
CRP	73.1 mg/L	<5 mg/L
Procalcitonin	2.95 ng/mL	<0.1 ng/mL
Glucose	6.7 mmol/L	3.9-6.1 mmol/L (fasting)
Troponin	<10 ng/L	<14 ng/L

He was admitted with the working diagnosis of a drug-related versus viral exanthem and treated with intravenous paracetamol, as-needed intravenous diphenhydramine, oral desloratadine, topical betamethasone, emollients, and calamine lotion. Over the next four days, his fever resolved, and the rash gradually improved, allowing for discharge on Day 4.

By Day 7 post-discharge, he developed gastrointestinal upset and presented to an outside clinic. A urea breath test confirmed Helicobacter pylori infection, for which he was prescribed triple therapy. On Day 12, after initiating this regimen, he experienced a severe relapse of rash and fever, prompting re-admission. On examination, the eruption was more intense, with confluent erythema and excoriations similar to the first presentation. The triple therapy was discontinued, and he was managed with supportive care. Importantly, systemic corticosteroids were not required during this admission, and his symptoms resolved with drug withdrawal, emollients, and antihistamines. Over the following days, his skin gradually improved, and he was discharged in stable condition. The absence of renal, hepatic, or cardiac organ involvement during both admissions was considered a favorable prognostic factor.

Following stabilization, he was reviewed in the rheumatology clinic for positive antinuclear antibody (ANA) and DFS70 antibody results, which were deemed likely false-positives as other autoimmune serologies were negative and lumbosacral radiography was unremarkable. These findings are summarized in Table 2.

**Table 2:** Autoimmune and immunology screening during follow-up. Autoimmune and immunology workup performed during follow-up revealed positive ANA and DFS70 antibody, with all other serologies negative. These findings were considered false positives in the clinical context, as there was no supportive evidence of systemic autoimmune disease. ANA, antinuclear antibody; DFS70 antibody, dense fine speckled 70-kDa autoantibody; C-ANCA, cytoplasmic antineutrophil cytoplasmic antibody; P-ANCA, perinuclear antineutrophil cytoplasmic antibody; SS-A antibody, anti-Sjögren’s-syndrome-related antigen A (Ro60); SS-B antibody, anti-Sjögren’s-syndrome-related antigen B (La); Scl-70 antibody, anti-topoisomerase I; Jo-1 antibody, anti-histidyl tRNA synthetase; Ro-52 antibody, anti-Ro52/TRIM21; PM-Scl antibody, anti-PM/Scl complex; CENP-B antibody, anti-centromere protein B; dsDNA, anti-double-stranded DNA; Nucleosomes, anti-nucleosome antibodies; Histones, anti-histone antibodies; Ribosomal P protein, anti-ribosomal P; AMA-M2, anti-mitochondrial antibody M2 subtype

Test	Result
ANA	Positive
DFS70 antibody	Positive
C-ANCA	Negative
P-ANCA	Negative
SS-A antibody	Negative
SS-B antibody	Negative
Scl-70 antibody	Negative
Jo-1 antibody	Negative
Ro-52 antibody	Negative
PM-Scl antibody	Negative
CENP-B antibody	Negative
dsDNA	Negative
Nucleosomes	Negative
Histones	Negative
Ribosomal P protein	Negative
AMA-M2	Negative

Two weeks later, he was assessed in the immunology clinic, where the diagnosis of DRESS syndrome was confirmed with a RegiSCAR score of 3, based on the presence of fever of 38.5 °C, a generalized morbilliform eruption with facial edema, peripheral eosinophilia (1.16 × 10⁹/L), a temporal relationship with recent antibiotic exposure, and the absence of an alternative infectious or autoimmune explanation on clinical and laboratory assessment [2]. He was counselled to permanently avoid vancomycin and all beta-lactams. The patient adhered well to follow-up recommendations, tolerated therapy without adverse effects, and remained clinically stable thereafter.

## Discussion

DRESS syndrome is a severe, potentially fatal idiosyncratic adverse drug reaction [12]. It typically begins with fever above 38 °C, followed by a generalized maculopapular or morbilliform exanthem, often starting on the face [1]. The rash may range from a mild exanthem to extensive erythroderma or exfoliative dermatitis, and facial edema is a characteristic finding that may predict more severe disease [5,9]. Rash is the most frequent clinical manifestation, present in 73%-100% of patients, with more than 50% of the body surface often affected [5,9]. Internal organ damage is common, most frequently involving the liver (75%), kidneys (37%), and lungs (32%), though cardiac, neurologic, gastrointestinal, and endocrine manifestations have also been described [2,12]. Lymphadenopathy is also reported in over half of patients [5].

Our patient presented in line with these classical features, with acute-onset fever, a rapidly spreading morbilliform rash, and facial edema. Notably, however, he did not demonstrate lymphadenopathy or visceral involvement, which are common in many reported cases. This absence of systemic organ dysfunction, along with his rapid clinical improvement, likely contributed to a more favorable prognosis. In contrast to many typical DRESS presentations, in which hepatic or renal injury and lymphadenopathy are common and often necessitate prolonged systemic corticosteroid therapy, our patient had a predominantly cutaneous phenotype without visceral involvement. Importantly, eosinophilia, which is sometimes absent in the early course [7], was present at initial admission in our patient, strengthening the suspicion of a drug-related hypersensitivity reaction. Despite the markedly elevated inflammatory markers, the lack of a clear infectious focus on history and examination, together with the close temporal relationship between antibiotic exposure and rash flares, supported DRESS over primary sepsis as the dominant process.

At the immunological level, DRESS is driven by activation of Th2 lymphocytes, which mediate type IVb hypersensitivity in the skin, while CD8+ T cells are responsible for internal organ injury [13]. Genetic susceptibility and impaired detoxification pathways contribute to risk [13]. Viral reactivation, particularly HHV-6 and EBV, has also been implicated, which is why the Japanese J-SCAR diagnostic criteria uniquely incorporate viral reactivation [14]. Although viral serology was not contributory in our case, the absence of systemic viral reactivation markers helped to narrow the diagnosis.

Given its heterogeneous and delayed presentation, diagnosis remains challenging. The European RegiSCAR scoring system provides a standardized framework, assessing clinical, laboratory, and organ features to establish the diagnosis [11]. It allocates points for fever, lymphadenopathy, eosinophilia, atypical lymphocytes, the extent of skin involvement, internal organ involvement, and exclusion of alternative causes, and then classifies cases as no, possible, probable, or definite DRESS [11]. Our patient’s diagnosis was confirmed with a RegiSCAR score of 3, consistent with a probable case. Importantly, the lack of mucosal involvement, visceral dysfunction, or lymphadenopathy reduced his overall score, despite the severity of the skin eruption. This case underscores the importance of applying structured diagnostic tools to avoid under-recognition of milder or atypical cases.

With respect to etiology, antiepileptics are the most frequently reported triggers [15]. However, clinicians should increasingly be aware of antibiotic-associated DRESS, as highlighted in recent reports [15]. Our case is significant because the patient initially developed a rash while on vancomycin and ceftriaxone and later experienced a more severe relapse following H. pylori triple therapy. This illustrates two critical points: (1) antibiotics can act as primary triggers, even after short courses, and (2) re-exposure to structurally different antimicrobials may precipitate recurrence, highlighting the need for extreme caution in future prescribing. Careful drug history and recognition of prior hypersensitivity were key in confirming the diagnosis and preventing further escalation. Because the initial rash occurred after several days of combined vancomycin and ceftriaxone therapy, it is not possible to attribute causality to a single agent with certainty. Nonetheless, this case adds to the growing body of reports implicating both glycopeptides and beta-lactam antibiotics in DRESS and illustrates how concurrent and sequential antibiotic exposures can complicate culprit identification in clinical practice.

Renal involvement, seen in up to 37% of cases and often linked to allopurinol or carbamazepine [5,12], was notably absent in our patient, who maintained normal creatinine and eGFR during both admissions. Similarly, hepatic injury, which represents the most common visceral complication, was limited to mild ALT elevation without clinically significant dysfunction. The absence of systemic organ involvement again underscores the favorable prognosis in this case, compared to more severe phenotypes with fulminant hepatitis or nephritis.

Interestingly, our patient’s autoimmune screen revealed positive ANA and DFS70 antibodies in the absence of other serological markers or clinical features. While these findings were interpreted as false positives, they are noteworthy in the context of DRESS, which carries a recognized long-term risk of autoimmune sequelae, including autoimmune thyroiditis, type 1 diabetes mellitus, systemic sclerosis, and systemic lupus erythematosus [9]. This highlights the importance of continued follow-up, as autoimmune complications may arise weeks to months after resolution of the acute illness.

The clinical course of our patient also illustrates that systemic corticosteroids, although considered the mainstay for severe cases, may not always be required. During his first admission, he received intravenous hydrocortisone, but during the relapse following H. pylori therapy, supportive care with antihistamines and emollients was sufficient for recovery. Corticosteroids were withheld during the relapse because he remained hemodynamically stable, had no evidence of organ dysfunction on serial laboratory testing, and his skin findings improved promptly after withdrawal of the suspected antibiotics and initiation of symptomatic treatment. This case therefore, supports a tailored approach, where corticosteroids are reserved for severe or systemic cases, while drug withdrawal and symptomatic therapy may suffice in milder relapses.

A limitation of this report is that detailed information on arterial blood gas analysis, chest imaging, viral studies, lymphocyte morphology, and histopathology is not included, although the diagnosis of DRESS was supported by the clinical course, characteristic laboratory abnormalities, and application of the RegiSCAR scoring system.

Taken together, this case contributes to the growing recognition of antibiotics as important culprits in DRESS. It emphasizes the utility of structured diagnostic scoring (RegiSCAR), the prognostic significance of organ involvement, and the need for long-term follow-up to monitor for autoimmune complications. Most importantly, it underscores the critical role of meticulous drug history and awareness of recurrence risk, which ultimately guided the diagnosis and management in our patient.

## Conclusions

DRESS syndrome remains a rare but potentially life-threatening drug reaction that requires early recognition and prompt discontinuation of the offending agent. This case highlights antibiotics as important, though less commonly recognized, culprits and illustrates the potential for recurrence following re-exposure, particularly in the setting of multiple antibiotic exposures. The absence of systemic organ involvement in our patient underscores the variability in clinical presentation and the possibility of favorable outcomes with timely intervention. Careful drug history, structured diagnostic assessment, individualized treatment according to organ involvement, and long-term follow-up are essential to monitor patient safety and detect late autoimmune sequelae. As a single case report, however, this description cannot determine recurrence rates or identify predictors of relapse, and the observations should be interpreted as hypothesis-generating rather than definitive.
